# The Effect of a Traditional Preparation Containing *Piper nigrum* L. and *Bunium persicum* (Boiss.) B.Fedtsch. on Immobility Stress-Induced Memory Loss in Mice

**DOI:** 10.1155/2021/5577594

**Published:** 2021-06-12

**Authors:** Marzieh Rashedinia, Mina Mojarad, Forouzan Khodaei, Ali Sahragard, Mohammad Javad Khoshnoud, Mohammad M. Zarshenas

**Affiliations:** ^1^Medicinal Plants Processing Research Center, Shiraz University of Medical Sciences, Shiraz, Iran; ^2^Department of Pharmacology Toxicology, School of Pharmacy, Shiraz University of Medical Sciences, Shiraz, Iran; ^3^College of Animal Science and Veterinary Medicine, Shanxi Agricultural University, Taigu, China; ^4^Food and Supplements Research Center, Shiraz University of Medical Sciences, Shiraz, Iran; ^5^Department of Phytopharmaceuticals (Traditional Pharmacy), School of Pharmacy, Shiraz University of Medical Sciences, Shiraz, Iran; ^6^Epilepsy Research Center, Shiraz University of Medical Sciences, Shiraz, Iran

## Abstract

**Objective:**

Alzheimer's disease is a progressive, age-related, and neurodegenerative disease characterized by mental decline. The exact cause of Alzheimer's disease is unclear, but cholinergic dysfunction, protein accumulation, and oxidative stress are among the most important hypotheses. The main purpose of our study was to investigate the effects of aqueous and hydroalcoholic extract combination of these two medicinal plants, black pepper and cumin (as a related formulation in traditional Persian medicine), on memory and learning of an immobilized stress animal model.

**Methods:**

In this study, hydroalcoholic and aqueous extracts of cumin and black pepper fruits were prepared. Six groups of mice were treated orally for 2 weeks: control group, immobility stress, and stress-induced immobility mice received different doses of the hydroalcoholic extract (100 and 200 mg/kg) and aqueous extract (100 and 200 mg/kg). The shuttle box, novel object detection, and rotarod test were used to evaluate memory and learning. The activities of acetylcholinesterase, catalase (CAT), and superoxide dismutase (SOD) and the level of reduced glutathione (GSH) and malondialdehyde (MDA) were measured in the brain tissue.

**Results:**

Immobility stress significantly reduced learning and motor coordination. Furthermore, MDA levels and acetylcholinesterase activity were significantly increased, while CAT and SOD activities were significantly reduced in the brain of immobility-induced stress mice. Other findings indicated that hydroalcoholic and aqueous extracts (100 and 200 mg/kg) of cumin and black pepper fruits have an improving effect on animal motor coordination and learning ability, GSH content, and CAT, SOD, and acetylcholinesterase enzyme function in comparison with stress groups (*p* < 0.05).

**Conclusion:**

The hydroalcoholic and aqueous extracts of cumin and black pepper fruits have protective effects against stress-induced memory deficit and oxidative stress and may have beneficial therapeutic effect in the treatment of neurodegenerative diseases.

## 1. Introduction

Alzheimer's disease is the most common progressive neurodegenerative disease and the most important cause of dementia in the elderly [1, 2]. Usually, the disease occurs in people over 65 years old, but less common cases start at young ages. It is estimated that more than 35 million people worldwide have Alzheimer's disease [3]. The exact cause of Alzheimer's disease is unclear, but cholinergic dysfunction and protein accumulation are among the most important hypotheses [4]. According to the cholinergic hypothesis, the use of acetylcholinesterase inhibitors alleviates the symptoms of Alzheimer's disease [5]. The protein accumulation hypothesis indicated that the accumulation of improperly folded proteins such as amyloid-beta (A*β*) and neurofibrillary tangle in the brain of people with Alzheimer's disease cause oxidative stress and inflammatory damage, which ultimately leads to synaptic dysfunction [6]. Reactive oxygen species (ROS) are free radicals, derived from oxygen, which have been shown to play a role in cell damage [7, 8]. They are produced naturally during mitochondrial respiration and energy production, but normally, they are neutralized by the cellular antioxidant defense systems [9–12].

In recent years, many studies have been conducted to develop promising drugs for treating Alzheimer's disease [13–15], but so far, no effective drugs have been developed. In traditional Persian medicine (TPM), Alzheimer's disease, as “*Nesiān*” (loss in memory), “*Farāmūshkārī*” (forgetfulness), and “*Fesād-e-Zekr*” (deterioration in memory), is defined as a difficulty in remembering recent events. Also, two related terms such as *Fesād-e-fekr* (deterioration in thought) and *Fesād-e-khial* (deterioration in imagination) have been mentioned in Persian manuscripts [16, 17].

In this investigation, a two-compartment formulation, with repeated citations in those encyclopedias, was selected from the third volume of *The Canon of Medicine* to enter the practical phases [18].

Cumin (*Bunium persicum* (Boiss.) B.Fedtsch.) is a plant from the Apiaceae family. Cumin is a crucial medicinal plant that is used to treat various diseases such as epilepsy, seizure, stomachache, bloating, and dyspepsia. Cumin contains 7.7% oil, 13.5% resin, gums, and mucus, and 15.5% protein [19–21].

Black pepper (*Piper nigrum* L.) is a plant from the *Piperaceae* family, a native plant in India, and is currently grown in other countries such as China [22]. Black pepper contains piperine, linoleic acid, oleic acid, and palmitic acid fatty acids. Piperine is one of the most effective ingredients in black pepper fruit. Oral use of piperine can reduce memory loss and hippocampal nerve damage in adult male Wistar rats. The anticholinergic and antioxidant properties of piperine have been reported [23, 24]. Black pepper can play an effective role in reducing damage to neurons and improving Alzheimer's disease through its antioxidant effect and inhibition of free radical production [25]. Therefore, based on the effects of these plants mentioned in traditional books and the pharmacological effects mentioned in modern medicine, the main purpose of our study was to investigate the effects of aqueous and hydroalcoholic extract combination of these two medicinal plants (black pepper and cumin) on memory and learning of an immobilized stress animal model. Also, the acetylcholinesterase (AChE) activity and oxidative stress indices were measured in the brain tissue of treated mice.

## 2. Materials and Methods

### 2.1. Chemicals and Reagents

EDTA, thiobarbituric acid (TBA), trichloroacetic acid (TCA), Na_2_HPO_4_, NaH_2_PO_4_, K_2_HPO_4_, and KH_2_PO_4_ were purchased from Merck (Darmstadt, Germany). 5,5′-Dithiobis-(2-nitrobenzoic acid), thioculin, and other chemicals were of the highest grade which were purchased from Sigma-Aldrich (St. Louis, Missouri, USA). Superoxide dismutase (SOD) and catalase (CAT) kits were purchased from NavandSalamat (Iran).

### 2.2. Preparation of Fruit Extracts

Wild-grown fruits of cumin and black pepper were purchased from the Medical Herbs Market in the southwest of Iran (Shiraz). After identifying and allocating the herbarium number, the fruits were dried in a shaded place at room temperature. The dose of extracts and their formulation have been selected based on the characteristics mentioned in *The Canon of Medicine* [18]. The mixture of cumin and black pepper fruits (1 : 1) was ground, and the hydroalcoholic and aqueous extracts were prepared using 70% ethanol and distilled water, respectively. Rapid extraction was done using sonication at 40°C for 20 min. The clear extract was separated using filter paper. This process was repeated three times, and the clear extracts were pooled. The extract was concentrated at 60°C using a rotary device and dried in a high-speed vacuum and freeze dryer. The amount of dried extract was weighed, and extraction efficiency was calculated.

### 2.3. Animal Treatment

Thirty-six mice weighing 25 ± 5 g were housed in special cages at 23 ± 2°C, 60% humidity, and 12 h light/dark cycle with food and water *ad libitum*. After one week of adaptation, the animals were randomly divided into six groups (6 in each group) including the control group, immobility stress group, immobility stress+100 mg/kg hydroalcoholic extract group, immobility stress+200 mg/kg hydroalcoholic extract group, immobility stress+100 mg/kg aqueous extract group, and immobility stress+200 mg/kg aqueous extract group. The restrainer was used to induce immobility stress [26]. The mice were placed inside the restrainers for two hours (from 9 a.m. to 11 a.m.) every day for two weeks. After the stress was over, the animals were returned to their cages, and after resting for half an hour, the extracts were administered by gavage. After behavioral tests, the animals were anesthetized by ketamine/xylazine (100/10 mg/kg, i.p.) and brain tissues were collected and stored at -70°C until biochemical analyses. The study was reviewed and approved by the Ethics Committee of the Shiraz University of Medical Sciences. All animal procedures were based on the *Guideline for the Care and Use of Laboratory Animals*.

### 2.4. Behavioral Tests

#### 2.4.1. Passive Avoidance Learning Test

The passive avoidance learning test was used to assess the effects of the treatment on the animals' learning and memory, according to the method previously described [27]. The shuttle box equipment used in this test consisted of a light chamber and a dark chamber with the same size and grid floor, separated by a movable guillotine door. For training, the mouse was placed in the lighted area; as soon as the mouse entered the dark area, the door was closed and the animal received an electrical foot shock (1 mA for 1 second). To assess the memory of the mice, the retention test was performed 24 h after the learning and the latency time to enter the dark area (step-through latency (STL)) and time spent in the dark compartment (TDC) were recorded up to 300 seconds.

#### 2.4.2. Novel Object Recognition Test

To assess recognition, the novel object test was used [28]. After the habituation of the animals in the box (50 cm × 50 cm × 50 cm), in the training phase, two equal objects were placed in the box for exploration by the mouse in 300 seconds. After 24 h, the testing phase started by exchanging one of the objects with a novel object. The time spent to explore the novel and familiar object was recorded within 300 seconds.

#### 2.4.3. Rotarod Test

The rotarod device was used to evaluate motor coordination. To evaluate the motor coordination, the animal stays on a rotating rod at 10 rpm. First, each animal is trained and tested after 24 hours. The time the animal remained on the rod was recorded in cut-off time 60 seconds [29].

### 2.5. Determination of Acetylcholinesterase Activity

The activity of acetylcholinesterase (AChE) was determined according to optimized Ellman's method [30]. In brief, the supernatant of homogenized brain tissues was incubated with DTNB as Ellman reagent and acetylthiocholine iodide as the substrate. The absorbance was measured at 412 nm by an automated plate reader (BioTek, Highland Park). The protein concentration was measured by the Bradford method [31], and AChE activity was expressed as U/mg protein.

### 2.6. Determination of Superoxide Dismutase and Catalase Activity

The activities of SOD and CAT were measured using a Nasdox and Nactaz kit (NavandSalamat, Iran), respectively, and according to the kit's instruction. The enzyme activities were expressed as U/mg protein [8, 32].

### 2.7. Measuring Glutathione Contents

The brain GSH content was measured as previously described [33]. In brief, 500 *μ*l of the brain homogenate was added to equal volume of TCA (10%), and the mixture was centrifuged at 4000 rpm for 10 min at 4°C. Then, 0.01 M DTNB and phosphate buffer (pH 8.9) were added to the supernatant. Absorption was measured by a plate reader (BioTek, Highland Park) at 412 nm. The protein concentration was measured by the Bradford method [31], and the GSH level was reported as *μ*M/mg protein.

### 2.8. Measuring Lipid Peroxidation Level

The brain homogenate (500 *μ*l) was mixed with 3 ml of 1% phosphoric acid and 1 ml of 0.6% thiobarbituric acid. The mixture was boiled in the water at 100°C for 45 min, cooled, and centrifuged at 10000 rpm for 10 min. Finally, the absorbance of the supernatant was measured at 532 nm. The protein concentration was measured by the Bradford method [31], and the level of MDA was expressed as *μ*M/mg protein [34].

### 2.9. Statistical Analysis

Statistical analyses were performed using GraphPad Prism software (version 6.0), and the data were reported as the mean ± SEM. One-way ANOVA followed by the Dunnett post hoc test was used to compare the variables between the groups. *p* < 0.05 was considered statistically significant.

## 3. Results

### 3.1. The Effects of Hydroalcoholic and Aqueous Extracts of Cumin and Black Pepper Fruit on the Behavioral Test

**Figure 1:**
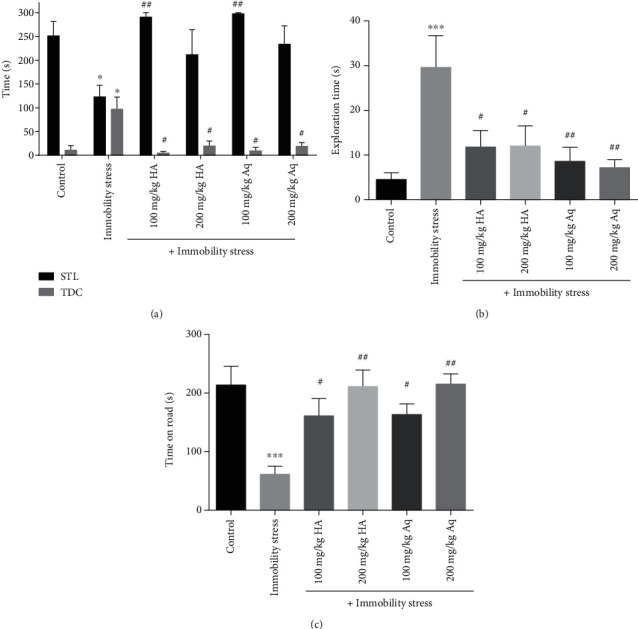
The effects of hydroalcoholic and aqueous extracts of cumin and black pepper fruits on the step-through latency (STL) and time spent in the dark compartment (TDC) in the shuttle box test (a). (b) The exploration time in the novel object detection test and (c) motor coordination. The values are mean ± SEM (*n* = 6). HA: hydroalcoholic extract; Aq: aqueous extract. ^∗^Significant difference compared to the control group (*p* < 0.05). ^∗∗∗^Significant difference compared to the control group (*p* < 0.001). ^#^Significant difference compared to the immobility stress group (*p* < 0.05). ^##^Significant difference compared to the immobility stress group (*p* < 0.01).

As shown in Figure 1(a), STL in the immobility stress group was reduced and the time spent in the dark compartment increased significantly compared with the control group (*p* < 0.05). In addition, treatment with hydroalcoholic (100 mg/kg) and aqueous (100 mg/kg) extract significantly improved STL and TDC compared to the immobility stress group (*p* < 0.01 and *p* < 0.05).

The result of the novel object detection test is illustrated in Figure 1(b). The time taken to identify the new object was significantly higher in the immobility stress group compared to the control group (*p* < 0.01). Treatment with both doses (100 and 200 mg/kg) of the hydroalcoholic and aqueous extract caused a significant decrease in the time taken to identify the new object when compared to the immobility stress group (*p* < 0.01, *p* < 0.05).

The results of the motor coordination in the rotarod test are shown in Figure 1(c). The falling latency in the immobility stress group was significantly lower than that in the control group (*p* < 0.01). The treatment with the hydroalcoholic and aqueous extract (100 and 200 mg/kg) caused a significant improvement of the latency to fall duration (*p* < 0.01, *p* < 0.05) compared with the immobility stress group.

### 3.2. The Effects of Hydroalcoholic and Aqueous Extracts of Cumin and Black Pepper Fruit on Brain Biochemical Factors

**Figure 2:**
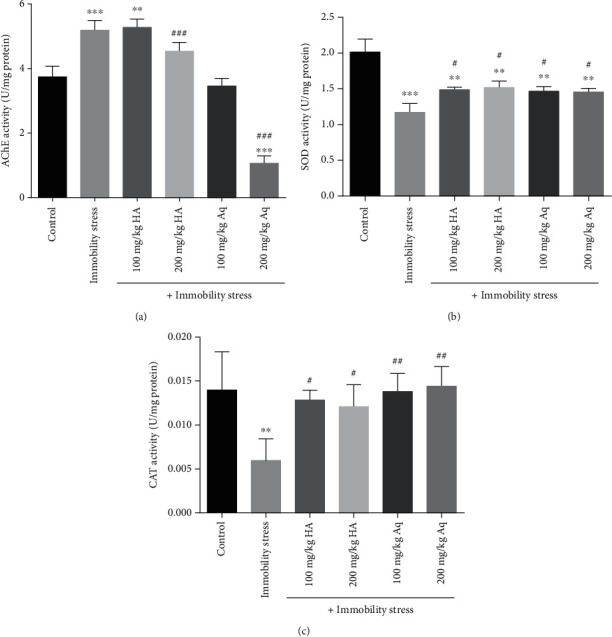
The effects of hydroalcoholic and aqueous extracts of cumin and black pepper fruits on (a) acetylcholinesterase (AChE) activity, (b) superoxide dismutase (SOD) activity, and (c) catalase (CAT) activity in the brain tissue. The values are mean ± SEM (*n* = 6). HA: hydroalcoholic extract; Aq: aqueous extract. ^∗∗^Significant difference compared to the control group (*p* < 0.01). ^∗∗∗^Significant difference compared to the control group (*p* < 0.001). ^#^Significant difference compared to the immobility stress group (*p* < 0.05). ^##^Significant difference compared to the immobility stress group (*p* < 0.01). ^###^Significant difference compared to the immobility stress group (*p* < 0.001).

As shown in Figure 2(a), immobility stress significantly increased brain AChE activity compared to the control group (*p* < 0.001). Treatment with aqueous extract (100 and 200 mg/kg) decreased AChE activity as compared with the immobility stress group (*p* < 0.001), but no significant differences in AChE activity were observed in hydroalcoholic extract (100 and 200 mg/kg)-treated animals compared with the immobility stress group.

A significant decrease in the brain SOD and CAT activities was observed (Figures 2(b) and 2(c)) after inducing immobility stress (*p* < 0.001 and *p* < 0.01, respectively). Also, the administration of the hydroalcoholic and aqueous extract (100 and 200 mg/kg) significantly increased the activities of SOD and CAT compared with the immobility stress group (*p* < 0.05 and *p* < 0.01, respectively).

**Figure 3:**
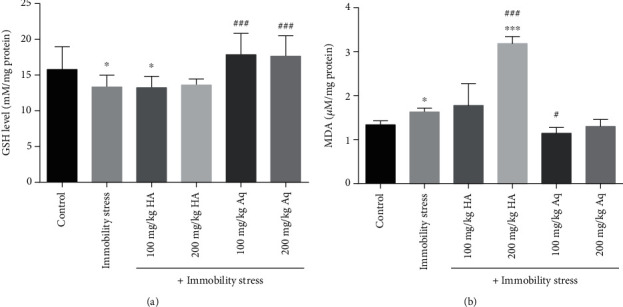
The effects of hydroalcoholic and aqueous extracts of cumin and black pepper fruits on (a) GSH content and (b) MDA level in the brain tissue. The values are mean ± SEM (*n* = 6). HA: hydroalcoholic extract; Aq: aqueous extract. ^∗^Significant difference compared to the control group (*p* < 0.05). ^∗∗∗^Significant difference compared to the control group (*p* < 0.001). ^#^Significant difference compared to the immobility stress group (*p* < 0.05). ^###^Significant difference compared to the immobility stress group (*p* < 0.001).

As shown in Figure 3(a), the immobility stress and hydroalcoholic extract (100 and 200 mg/kg) significantly reduced the GSH content of the brain (*p* < 0.05). The GSH content of the brain was significantly increased (*p* < 0.001) in the animals treated with the aqueous extract (100 and 200 mg/kg) compared with the immobility stress group. As shown in Figure 3(b), the brain MDA level was significantly increased in the immobility stress and hydroalcoholic (200 mg/kg) groups compared with the control group (*p* < 0.05 and *p* < 0.001, respectively), while the administration of aqueous extract (100 mg/kg) significantly reduced the brain MDA compared with the immobility stress group (*p* < 0.05).

## 4. Discussion

Previous studies have indicated that stress causes depression, anxiety, and mood disorders [35]. Also, stress harms cognitive functions by increasing the secretion of glucocorticoids that activate the hypothalamus-pituitary-adrenal axis [26]. Stressful situations cause cortisol hormone release from the adrenal cortex which easily passes the blood-brain barrier. Also, the performance of memory areas including the frontal cortex, hippocampus, and amygdala is affected by the release of this hormone, due to the presence of glucocorticoid receptors in these areas [35]. In the present study, the effect of hydroalcoholic and aqueous extracts of cumin and black pepper on learning and memory impairment induced by immobility stress was investigated. Results indicated that immobility stress impaired memory function by shortening the STL in the shuttle box and exploration time in the novel object test. Our data also demonstrated that treatment with cumin and black pepper extract has potent memory-enhancing effects on animal behavioral tests. Piperine is one of the most effective ingredients in black pepper fruit, which have revealed considerable improvements in memory loss in previous animal model studies [36, 37]. The possible mechanism of this effect has suggested the improvement of neural density and cholinergic function in the hippocampus [38]. In our study, acute stress also reduced the coordination in the rotarod test which is in agreement with the results of previous studies that demonstrated ameliorating effects of piperine treatment on motor coordination and cognitive dysfunction in an animal mouse model of Parkinson's disease [39]. Various studies on animal models of acute and chronic stress have showed oxidative damage in the brain as a result of the overproduction of reactive oxygen species [40, 41]. ROS reacts with biological molecules, including cellular lipids, proteins, and nucleic acids [42], reducing cellular redox and antioxidant defense of neuronal cells leading to apoptosis and eventually impaired memory and learning functions [43, 44]. So, using natural compounds that may have beneficial antioxidant effects in neurodegenerative disease is considered a new approach to improve cognitive and behavioral disorders as well as enhance memory [45, 46]. In our study, animal treatment with cumin and black pepper aqueous extracts significantly attenuated reduced MDA level as well as increased GSH content and SOD and CAT activities in the brain. This may be due to the antioxidant properties of piperine and cumin, as previously reported [36, 47]. Besides oxidative stress, inflammatory processes play an important role in the pathogenesis of cognitive impairment associated with neurodegenerative diseases [48]. Roshanbakhsh et al. indicated that piperine enhanced antioxidant capacity, inhibited iNOS, and reduced TNF-*α*, IL1-*β*, and NF-*κ*B expression levels, in the hippocampal tissue [37]. Owing to the anti-inflammatory activity of piperine, anti-inflammatory mechanisms may also be involved in the observed black pepper activity, although further investigation is necessary to characterize the active constituent(s) of cumin and black pepper responsible for such activities.

The pharmacological activities of cumin on cognition and memory were less studied than piperine. Inconsistent with our results, a previous study demonstrated the antioxidant, antistress, and memory-enhancing activities of cumin aqueous extract in animals. The authors suggested that central cholinomimetic activity and free radical scavenging mechanisms of cumin enhanced memory in scopolamine-induced memory loss in rats [49]. Based on our GSH and MDA results, probably aqueous extracts of cumin and black pepper are more effective than hydroalcoholic extract. Previously, Koppula et al. described that up to one-week treatment with aqueous extracts of *Cuminum cyminum* did not have effects on TBARs of the brain and liver tissue of rats.

Brain or other tissue toxicity may occur because the selected doses in our study are too high, especially in the hydroalcoholic extract. Increasing lipid peroxidation in the brain of the animals treated with hydroalcoholic extract may be due to increasing the concentration of piperine, cumin, or other active ingredients in the hydroalcoholic extract in comparison with the aqueous extract. One of the limitations of our study is the lack of high-dose treatment of aqueous and hydroalcoholic extract individually to evaluate the toxicity of each one.

In all, the mixture of cumin and black pepper is used in Iranian traditional medicine to reduce anxiety and produce calming effects. The hydroalcoholic and aqueous extracts of cumin and black pepper fruits have protective effects against stress-induced memory deficit and oxidative stress and may have beneficial therapeutic effect in the treatment of neurodegenerative diseases. Further research is required to reveal the mechanism of neuroprotective effects.

## Data Availability

All related data are in the manuscript.
